# A spatial emergent constraint on the sensitivity of soil carbon turnover to global warming

**DOI:** 10.1038/s41467-020-19208-8

**Published:** 2020-11-02

**Authors:** Rebecca M. Varney, Sarah E. Chadburn, Pierre Friedlingstein, Eleanor J. Burke, Charles D. Koven, Gustaf Hugelius, Peter M. Cox

**Affiliations:** 1 College of Engineering, Mathematics and Physical Sciences, University of Exeter, Laver Building, North Park Road, Exeter, EX4 4QF UK; 2 LMD/IPSL, ENS, PSL Université, École Polytechnique, Institut Polytechnique de Paris, Sorbonne Université, CNRS, 75006 Paris, France; 3 Met Office Hadley Centre, FitzRoy Road, Exeter, EX1 3PB UK; 4 Earth and Environmental Sciences Division, Lawrence Berkeley National Laboratory, Berkeley, CA 94720 USA; 5 Department of Physical Geography and Bolin Centre of Climate Research, Stockholm University, Stockholm, 10691 Sweden

**Keywords:** Climate and Earth system modelling, Projection and prediction

## Abstract

Carbon cycle feedbacks represent large uncertainties in climate change projections, and the response of soil carbon to climate change contributes the greatest uncertainty to this. Future changes in soil carbon depend on changes in litter and root inputs from plants and especially on reductions in the turnover time of soil carbon (*τ*_s_) with warming. An approximation to the latter term for the top one metre of soil (Δ*C*_s,τ_) can be diagnosed from projections made with the CMIP6 and CMIP5 Earth System Models (ESMs), and is found to span a large range even at 2 °C of global warming (−196 ± 117 PgC). Here, we present a constraint on Δ*C*_s,τ_, which makes use of current heterotrophic respiration and the spatial variability of *τ*_s_ inferred from observations. This spatial emergent constraint allows us to halve the uncertainty in Δ*C*_s,τ_ at 2 °C to −232 ± 52 PgC.

## Abstract (web summary)

The fate of the carbon locked away in soil is uncertain, and there are vast differences between models. Here the authors apply observational, spatio-temporal constraints on carbon turnover projections and find that uncertainty in estimations of carbon dynamics are reduced by 50%.

## Introduction

**Fig. 1:**
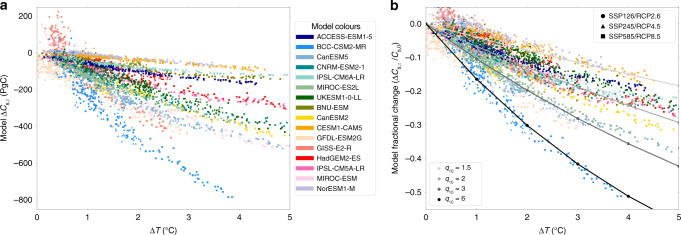
Uncertainty in future changes in soil carbon due to reduction in turnover time. Δ*C*_s,τ_ vs. Δ*T* plot diagnosed from sixteen Earth System Models (seven CMIP6 ESMs and nine CMIP5 ESMs), for three different future scenarios: SSP126, SSP245, SSP585, or RCP2.6, RCP4.5, RCP8.5, respectively. **a** The change in soil carbon due to the change in soil carbon turnover time against change in global mean temperatures; **b** The fractional change in soil carbon due to the change in soil carbon turnover time against change in global mean temperatures, and compared to different effective q_10_ sensitivities.

Climate–carbon cycle feedbacks^1^ must be understood and quantified if the Paris Agreement Targets are to be met^2^. Changes in soil carbon represent a particularly large uncertainty^3–7^, with the potential to significantly reduce the carbon budget for climate stabilisation at 2 °C global warming^8^. Previous studies have investigated the response of soil carbon to climate change based on both observational studies^9^ and Earth System Models (ESMs)^10^. ESMs are coupled models which simulate both climate and carbon cycle processes. Projects such as the Coupled Model Inter-comparison Project (CMIP)^11,12^, have allowed for consistent comparison of the response of soil carbon under climate change from existing state-of-the-art ESMs. However, the uncertainty due to the soil carbon feedback did not reduce significantly between the CMIP3 and CMIP5 model generations^6^, or with the latest CMIP6 models (see Fig. 1 and Supplementary Fig. 1), such that the projected change in global soil carbon still varies significantly amongst models^13^.

This study uses an alternative method to obtain a constraint on the ESM projections of soil carbon change. In previous studies, emergent constraints based on temporal trends and variations have been used successfully to reduce uncertainty in climate change projections^14^. Our approach follows the method used in Chadburn et al.^15^, where a spatial temperature sensitivity is used to constrain the future response to climate change—which we term as a *spatial emergent constraint*. Our study combines the Chadburn et al.^15^ method with the soil carbon turnover analysis of Koven et al.^16^ to get a constraint on the sensitivity of soil carbon turnover to global warming.

Soil carbon (*C*_s_) is increased by the flux of organic carbon into the soil from plant litter and roots, and decreased by the breakdown of that organic matter by soil microbes which releases CO_2_ to the atmosphere as the heterotrophic respiration flux (*R*_h_). If the vegetation carbon is at steady-state, litter-fall will equal the Net Primary Production of plants (NPP). If the soil carbon is also near to a steady-state—and in the absence of significant fire fluxes and other non-respiratory carbon losses—the litter-fall, NPP, and *R*_h_ will be approximately equal to one another. Even over the historical period, when atmospheric CO_2_ has been increasing and there has been a net land carbon sink, this approximation holds well (see Supplementary Fig. 4).

In order to separate the effects of changes in NPP from the effects of climate change on *R*_h_, we define an effective turnover time^17^ for soil carbon as *τ*_s_ = *C*_s_/*R*_h_. The turnover time of soil carbon is known to be especially dependent on temperature^3^. A common assumption is that *τ*_s_ decreases by about 7% per °C of warming (equivalent to assuming that q_10_ = 2)^18^. However, this sensitivity differs between models, and also between models and observations.

We can write a long-term change in soil carbon (Δ*C*_s_), as the sum of a term arising from changes in litter-fall (Δ*C*_s,L_), and a term arising from changes in the turnover time of soil carbon (Δ*C*_s,τ_):

1 $$\Delta {C}_{{\rm{s}}}= \Delta {\rm{(}}{R}_{{\rm{h}}}\ {\tau }_{{\rm{s}}}{\rm{)}}\approx \Delta {C}_{{\rm{s,L}}}{\rm{(t)}}+\Delta {C}_{{\rm{s,\tau }}}{\rm{(t)}} = {\tau }_{{\rm{s,0}}}\ \Delta {R}_{{\rm{h}}}{\rm{(t)}}+{R}_{{\rm{h,0}}}\ \Delta {\tau }_{{\rm{s}}}{\rm{(t)}}$$

Model projections of the first term (Δ*C*_s,L_) differ primarily because of differences in the extent of CO_2_-fertilisation of NPP, and associated nutrient limitations. The second term (Δ*C*_s,τ_) differs across models because of differences in the predicted future warming, and because of differences in the sensitivity of soil carbon decomposition to temperature (which includes an influence from faster equilibration of fast-turnover compared to slow-turnover carbon pools under changing inputs^13^). This study provides an observational constraint on the latter uncertainty. As the vast majority of the CMIP6 and CMIP5 models do not yet represent vertically resolved deep soil carbon in permafrost or peatlands, we focus our constraint on carbon change in the top 1 metre of soil. To ensure a fair like-for-like comparison we also exclude the two CMIP6 models that do represent vertically-resolved soil carbon (CESM2 and NorESM2), although this has a negligible effect on our overall result. Our study therefore applies to soil carbon loss in the top 1 metre of soil only. Below we show that it is possible to significantly reduce the uncertainty in this key feedback to climate change using current-day spatial data to constrain the sensitivity to future warming.

## Results and discussion

### Proof of concept

**Fig. 2:**
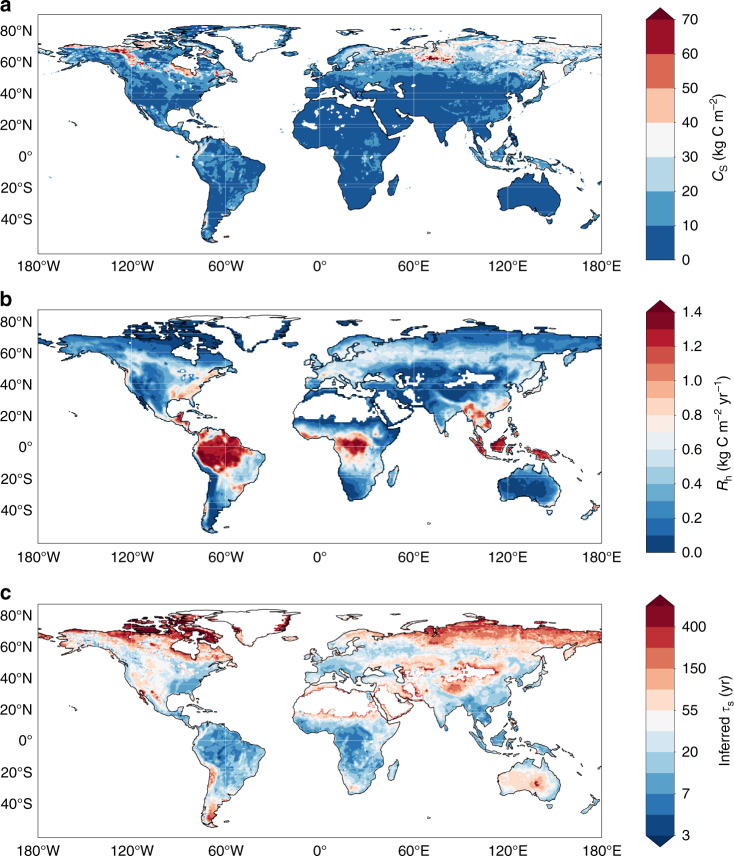
Spatial variability of soil carbon turnover time inferred from observations. Maps of **a** observed soil carbon (*C*_s_) to a depth of 1 m (kg C m^−2^)^19,20^, **b** observed heterotrophic respiration (*R*_h,0_) (kg C m^−2^ yr^−1^)^21^, and **c** inferred soil carbon turnover time ($\mathrm{log}\,{\tau }_{{\rm{s}}}$) (yr).

For each ESM, we begin by calculating the effective *τ*_s_ using time-averaged (1995–2005) values of *C*_s_ and *R*_h_ at each grid-point, and applying our definition of *τ*_s_ = *C*_s_/*R*_h_. We do likewise for observational datasets of soil carbon in the top 1 metre^19,20^ and time-averaged (2001–2010) heterotrophic respiration^21^, as shown in Fig. 2. Figure 2c shows the map of inferred values of *τ*_s_ from these observations, with a notable increase from approximately 7 years in the warm tropics to over 100 years in the cooler high northern latitudes.

Similar maps can be diagnosed for each of year of data, for each ESM, and for each future scenario, giving time and space varying values of *τ*_s_ for each model run. This allows us to estimate Δ*C*_s,τ_, via the last term on the right of Eq. (1). For each ESM, the *R*_h,0_ value is taken as the mean over the decade 1995–2005, to overlap with the time period of the observations and to maintain consistency across CMIP generations. Individual grid-point *τ*_s_ values are calculated for each year before calculating area-weighted global totals of Δ*C*_s,τ_. The uncertainty of Δ*C*_s,τ_ stems from the uncertainty in soil carbon turnover (*τ*_s_), and the uncertainty due to differing climate sensitivities of the models. In this study, we aim to quantify and constrain the uncertainty in *τ*_s_. To isolate the latter uncertainty, we consider Δ*C*_s,τ_ for differing levels of global mean warming in each model. The resulting dependence of global total Δ*C*_s,τ_ on global warming is shown in Fig. 1a, for each of the ESMs considered in both CMIP6 and CMIP5 (seven CMIP6 ESMs and nine CMIP5 ESMs), and for three Shared Socioeconomic Pathways (SSP): SSP126, SSP245 and SSP585 (CMIP6)^22^, or the equivalent Representative Concentration Pathways (RCP): RCP2.6, RCP4.5 and RCP8.5 (CMIP5)^23^. In all cases Δ*C*_s,τ_ is negative, which is consistent with the soil carbon turnover time decreasing with warming. The more surprising thing to note is the huge range in the projections, with a spread at 2 °C global mean warming of approximately 400 PgC, regardless of future SSP/RCP scenario. Figure 1b plots the fractional change in soil carbon Δ*C*_s,τ_/*C*_s,0_, showing that there is a large range of effective q_10_ sensitivities between the model projections.

Unfortunately, we do not have time-varying observational datasets of *C*_s_ and *R*_h_ that might allow us to directly constrain this projection uncertainty. Instead we explore whether the observed spatial variability in *τ*_s_ (as shown in Fig. 2c) provides some observational constraint on the sensitivity of *τ*_s_ to temperature. In doing so, we are motivated by Chadburn et al.^15^ who used the correlation between the observed geographical distributions of permafrost and air temperature to constrain projections of future permafrost area under global warming. Similarly, we use ESMs to test whether the spatial variation in *τ*_s_ reveals the sensitivity of soil carbon turnover to temperature. The spatial patterns of *τ*_s_ in CMIP5 simulations and observations were previously shown in Koven et al.^16^, and here we test whether such relationships can be used to estimate the response of soil carbon to future climate change, using a combination of CMIP6 and CMIP5 models.

**Fig. 3:**
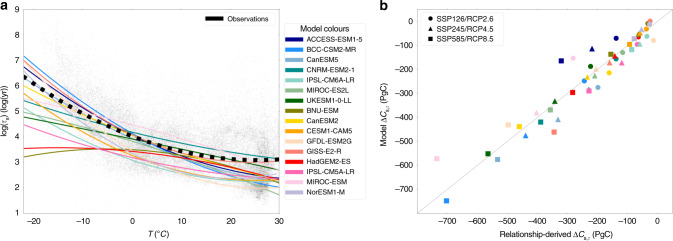
Using spatial variability of soil carbon turnover time to estimate Δ*C*_s,τ_. **a** Scatter-plot of the relationship between $\mathrm{log}\,{\tau }_{{\rm{s}}}$ and mean air temperature from observations^19–21,24^ (black points), and a quadratic fit (black-dotted line) representing the observational temperature sensitivity of $\mathrm{log}\,{\tau }_{{\rm{s}}}$. The equivalent quadratic fits for the ESMs are shown by the coloured lines; **b** The proof of principle for our method, showing an actual vs. estimated comparison, representing the modelled versus the relationship-derived values of the Δ*C*_s,τ_, where the change is considered between the start (1995–2005) and the end (2090–2100) of 21st century and is assumed to relate to the top 1 metre of soil.

Figure 3a is a scatter plot of $\mathrm{log}\,{\tau }_{{\rm{s}}}$ against temperature, using the *τ*_s_ values shown in Fig. 2c and mean temperatures from the WFDEI dataset over the period 2001–2010^24^. The thick black-dotted line is a quadratic fit through these points. Also shown for comparison are equivalent quadratic fits for each model (coloured lines), using the model $\mathrm{log}\,{\tau }_{{\rm{s}}}$ and mean near-surface air temperature (*T*) values for each grid-point, over an overlapping period with the observations (1995–2005). There is a spread in the individual data points due to variation in soil moisture, soil type, and other soil parameters^25^. The model specific spread in the data can be seen for the CMIP6 and CMIP5 models in Supplementary Figs. 2 and 3, respectively. Although models do not account for every possible factor contributing to this spread, the spread of points in the models is generally similar to the observations. However, differences between the best-fit functions relating *τ*_s_ to *T* are evident between the models, and between the models and the observations^16^.

This suggests that we may be able to constrain Δ*C*_s,τ_ using the observed *τ*_s_ vs. *T* fit from the observations, but only if we can show that such functions can be used to predict Δ*C*_s,τ_ under climate change. In order to test that premise, we attempt to reconstruct the time-varying Δ*C*_s,τ_ projection for each model using the time-invariant *τ*_s_ vs. *T* fit across spatial points (Fig. 3a), and the time-invariant *R*_h,0_ field. The change in soil carbon turnover time (Δ*τ*_s_(t)) for a given model run is estimated at each point based-on the *τ*_s_ vs. *T* curve, and the time-varying projection of *T* at that point. A local estimate of the subsequent change in soil carbon can then be made based-on the farthest right-hand term of Eq. (1) (*R*_h,0_ Δ*τ*_s_), which can be integrated up to provide an estimated change in global soil carbon in the top 1 metre (Δ*C*_s,τ_).

Figure 3b shows the result of this test for all models and all respective SSP/RCP scenarios. The axes of this plot show equivalent variables which represent the global Δ*C*_s,τ_ between the mean value for 2090–2100 and the mean value for 1995–2005. The *y*-axis represents the actual values for each model as shown in Fig. 1, and the *x*-axis represents our estimate derived from spatial variability (as in Fig. 3a). As hoped, actual vs. estimated values cluster tightly around a one-to-one line with an *r*^2^ correlation coefficient value of 0.90. Although some hot-climate regions will inevitably experience temperatures beyond those covered by current-day spatial variability, these tend to be regions with low soil carbon, so this does not have a major impact on the success of our method.

### Spatial emergent constraint

**Fig. 4:**
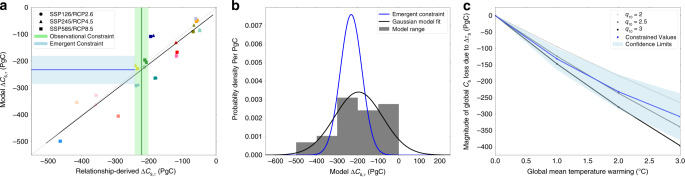
Emergent constraint on Δ*C*_s,τ_ as a function of global warming. **a** Actual vs. estimated scatter plot for Δ*C*_s,τ_ for 2 °C of global warming. The vertical green line defines the observational constraint which is derived using observational data and the future spatial temperature field of each model (decadal average), and the shaded region represents the corresponding uncertainty (±1 standard deviation). The horizontal blue line represents our emergent constraint, with the shaded region showing the corresponding uncertainty (±1 standard deviation) which results from the differing future spatial warming patterns seen in the future spatial temperature fields across the ESMs, and the emergent relationship between the model data points (black line). **b** Probability density function showing the Gaussian distribution of Δ*C*_s,τ_ values from the unweighted prior model ensemble (black line) and the emergent constraint (blue line). **c** Constrained Δ*C*_s,τ_ values at different levels of global warming (blue), including the likely (±1 standard deviation) uncertainty bounds (shaded blue). Different effective global q_10_ values shown for comparison; our emergent constraint is consistent with an effective q_10_ ≈ 2.5 ± 0.6.

This gives us confidence to use the *τ*_s_ vs. *T* fit and *R*_h,0_ from observations to constrain future projections of Δ*C*_s,τ_. To remove the uncertainty in future Δ*C*_s,τ_ due to the climate sensitivity of the models, we investigate a common amount of global mean warming in each model. Figure 4a is similar to Fig. 3b but instead for the more policy-relevant case of 2 °C of global warming. As before, the *y*-axis represents the modelled Δ*C*_s,τ_, and the *x*-axis is our estimate derived from spatial variability. Once again, the actual and estimated values of Δ*C*_s,τ_ cluster around the one-to-one line (with *r*^2^ = 0.87). The model range arises partly from differences in the initial field of heterotrophic respiration (*R*_h,0_), and partly from differences in Δ*τ*_s_ (compare first row to penultimate row of Table 1).

**Table 1:** Sensitivity study of spatial emergent constraint.

Constrained Δ*C*_s,τ_ at 2 °C global mean warming			
	Combined	CMIP6	CMIP5
CARDAMOM *R*_h_	−232 ± 52	−238 ± 62	−227 ± 48
MODIS NPP	−201 ± 53	−206 ± 63	−196 ± 49
Raich 2002 *R*_s_	−243 ± 50	−249 ± 59	−238 ± 46
CARDAMOM *R*_h_ (Observational *τ*_s_ v *T* fit, model *R*_h,0_)	−227 ± 95	−220 ± 75	−230 ± 109
Unconstrained Δ*C*_s,τ_	−196 ± 117	−216 ± 109	−180 ± 121

The table presents the sensitivity of the emergent constraint on Δ*C*_s,τ_ to model ensemble: CMIP5, CMIP6 or CMIP5 and CMIP6 combined (columns), and to the observational dataset for heterotrophic respiration (rows). The penultimate row presents the constraint using the observational *τ*_s_ v T fit and model *R*_h,0_, opposed to observational *R*_h,0_, to isolate the uncertainty reduction from these different components. For comparison, the last row shows the mean and standard deviation of the unconstrained model ensemble.

The vertical green line in Fig. 4a represents the mean estimate when the *τ*_s_ vs. *T* relationship and the *R*_h,0_ field from the model are replaced with the equivalents from the observations. The spread shown by the shaded area represents the relatively small impact on Δ*C*_s,τ_ of differences in modelled spatial climate change patterns at 2 °C of global warming. In order to estimate the remaining uncertainty in Δ*C*_s,τ_, we treat this spread as equivalent to an observational uncertainty in an emergent constraint approach^26^. We apply a standard statistical approach^27,28^ to estimate the probability density function of the *y*-axis variable (model Δ*C*_s,τ_), accounting for both this observational spread and the quality of the emergent relationship. To test the robustness to the choice of observations we have repeated the analysis with different datasets that represent heterotrophic respiration, which produces strongly-overlapping emergent constraints, and completing the analysis with both CMIP6 and CMIP5 models shows that the result is also robust to the choice of model ensemble (see Table 1).

Figure 4b shows the resulting emergent constraint (blue line), and compares to the unweighted histogram of model values (grey blocks), and a Gaussian fit to that prior distribution (black line). The spatial emergent constraint reduces the uncertainty in Δ*C*_s,τ_ at 2 °C of global warming from −196 ± 117 PgC to −232 ± 52 PgC (where these are mean values plus and minus one standard deviation for the top 1 metre). This same method can be applied to find constrained values of Δ*C*_s,τ_ for other values of global warming. Figure 4c shows the constrained range of Δ*C*_s,τ_ as a function of global warming. This rules out the most extreme projections but nonetheless suggests substantial soil carbon losses due to climate change even in the absence of losses of deeper permafrost carbon.

## Methods

### Obtaining spatial relationships

In this section we explain how the quadratic relationships representing the spatial $\mathrm{log}\,{\tau }_{{\rm{s}}}$-temperature sensitivity shown in Fig. 3a (and Supplementary Figs. 2, 3 and 6) were derived, for both the Earth System Models (ESMs) in CMIP6 and CMIP5, and using the observational data. This is similar to the method used in Koven et al.^16^.

### Obtaining spatial relationships for CMIP models

The CMIP6 models used in this study are shown in the Table 2, and the CMIP5 models used in this study are shown in Table 3.

**Table 2:** CMIP6 models.

Model	Institute
ACCESS-ESM1-5	Australian Community Climate and Earth Systems Simulator, Australia
BCC-CSM2-MR	The Beijing Climate Center, China
CanESM5	Canadian Centre for Climate Modelling and Analysis, Canada
CNRM-ESM2-1	CNRM/CERFACS, French Centre National de la Recherche Scientifique, France
IPSL-CM6A-LR	Institut Pierre-Simon Laplace, France
MIROC-ES2L	Atmosphere and Ocean Research Institute
	and Japan Agency for Marine-Earth Science and Technology, Japan
UKESM1-0-LL	NERC and Met Office Hadley Centre, UK

**Table 3:** CMIP5 models.

Model	Institute
BNU-ESM	College of Global Change and Earth System Science, China
CanESM2	Canadian Centre for Climate Modelling and Analysis, Canada
CESM1-CAM5	National Science Foundation, Department of Energy, NCAR, USA
GFDL-ESM2G	NOAA Geophysical Fluid Dynamics Laboratory, USA
GISS-ES-R	NASA Goddard Institute for Space Studies, USA
HadGEM2-ES	Met Office Hadley Centre, UK
IPSL-CM5A-LR	Institut Pierre-Simon Laplace, France
MIROC-ESM	Atmosphere and Ocean Research Institute and
	Japan Agency for Marine-Earth Science and Technology, Japan
NorESM-M	Norwegian Climate Centre, Norway

To obtain model specific spatial $\mathrm{log}\,{\tau }_{{\rm{s}}}$-temperature relationships, the following method was used. A reference time period was considered (1995–2005), this was taken as the end of the CMIP5 historical simulation to be consistent across CMIP generations and to best match the observational data time frame considered. Then, monthly model output data was time averaged over this period, for the output variables ‘soil carbon content’ (*C*_s_) in kg m^−2^, ‘heterotrophic respiration carbon flux’ (*R*_h_) in kg m^−2^s^−1^, and ‘air temperature’ in K. The variables *C*_s_ and *R*_h_ were used to obtain values for soil carbon turnover time (*τ*_s_) in years, using the equation *τ*_s_ = *C*_s_/(*R*_h_ × 86400 × 365). The model temperature variable units were converted from K to °C.

For each model, these values of $\mathrm{log}\,{\tau }_{{\rm{s}}}$ were plotted against the corresponding spatial temperature data to obtain the spatial $\mathrm{log}\,{\tau }_{{\rm{s}}}$-temperature plot. Then, quadratic fits (using the python package numpy polyfit) are calculated for each model, which represent the spatial $\mathrm{log}\,{\tau }_{{\rm{s}}}$ relationship and sensitivity to temperature. These model specific relationships are shown by the coloured lines in Fig. 3a in the main manuscript, and in Supplementary Fig. 2 for CMIP6 and in Supplementary Fig. 3 for CMIP5.

### Obtaining spatial relationships for observations

Following Koven et al.^16^, we estimated observational soil carbon data (to a depth of 1 m) by combining the Harmonized World Soils Database (HWSD)^19^ and Northern Circumpolar Soil Carbon Database (NCSCD)^20^ soil carbon datasets, where NCSCD was used where overlap occurs. To calculate soil carbon turnover time, *τ*_s_, using the following equation: *τ*_s_ = *C*_s_/*R*_h_, we require a global observational dataset for heterotrophic respiration. In the main manuscript, CARDAMOM (2001–2010) heterotrophic respiration (*R*_h_) is used^21^. We completed a sensitivity study on the choice of observational heterotrophic respiration dataset, see below. The WFDEI dataset is used for our observational air temperatures (2001–2010)^24^. Then, these datasets can be used to obtain the observational $\mathrm{log}\,{\tau }_{{\rm{s}}}$-temperature relationship, using the same quadratic fitting as with the models. This represents the ‘real world’ spatial temperature sensitivity of $\mathrm{log}\,{\tau }_{{\rm{s}}}$, and is shown by the thick-dotted-black line in Fig. 3a of the main manuscript. A comparison of the derived observational relationships can be seen in Supplementary Fig. 6.

### Observational sensitivity study

We completed a sensitivity study to investigate our constraint dependence on the choice of observational heterotrophic respiration dataset (CARDAMOM (2001–2010)^21^). The other observational datasets considered are as follows: NDP-08 ‘Interannual Variability in Global Soil Respiration on a 0.5 Degree Grid Cell Basis’ dataset (1980–1994)^29^, ‘Global spatiotemporal distribution of soil respiration modelled using a global database’^30^, and MODIS net primary productivity (NPP) (2000–2014)^31^. Supplementary Fig. 4 shows scatter plots showing one-to-one comparisons of these observational datasets against one another, and Supplementary Fig. 5 shows the corresponding comparisons of the equivalent $\mathrm{log}\,{\tau }_{{\rm{s}}}$ values calculated from each dataset.

The CARDAMOM *R*_h_ dataset is used in the main manuscript for the following two main reasons: firstly, we calculate *τ*_s_ using heterotrophic respiration which allows for consistency between models and observations, and secondly, the dataset does not use a prescribed q_10_ sensitivity^21^. Instead, the CARDAMOM *R*_h_ dataset was derived by explicitly assimilating observations into a process-based diagnostic land-surface model. To test the robustness of our results, we also repeated our analysis with MODIS NPP and Raich 2002, for both CMIP6 and CMIP5 together, and as separate model ensembles. Supplementary Fig. 6 shows the observational $\mathrm{log}\,{\tau }_{{\rm{s}}}$-temperature relationships, derived using each of these observational datasets. The results are presented in Table 1 which shows the constrained values of Δ*C*_s,τ_ at 2 °C global mean warming.

We decided not to complete the paper analysis using the Hashimoto dataset since not only is it inconsistent with the three other datasets considered, it also shows an arbitrary maximum respiration level (Supplementary Fig. 4), which likely results from the assumed temperature-dependence of soil respiration in this dataset which takes a quadratic form^30^. The quadratic form is justified based on a site-level study in which it is used to fit temporal dynamics. However, the parameters for the quadratic function that are fitted in the Hashimoto study are very different from those in the site-level study, which therefore suggests that the same relationship does not apply to the global distribution of mean annual soil respiration.

### Equation for the soil carbon turnover time component of soil carbon change

The equation used in this study for the component of the change in soil carbon (Δ*C*_s_) due to the change in soil carbon turnover time (Δ*τ*_s_) was derived in the following way. Starting with the equation for soil carbon (based on the definition of *τ*_s_):

2 $${C}_{{\rm{s}}}={R}_{{\rm{h}}}\ {\tau }_{{\rm{s}}}$$

As discussed in the main manuscript, we can write this change in soil carbon (Δ*C*_s_), as the sum of a term arising from changes in litter-fall (Δ*C*_s,L_), and a term arising from changes in the turnover time of soil carbon (Δ*C*_s,τ_):

3 $$\Delta {C}_{{\rm{s}}}= \Delta {\rm{(}}{R}_{{\rm{h}}}\ {\tau }_{{\rm{s}}}{\rm{)}}\approx \Delta {C}_{{\rm{s,L}}}{\rm{(t)}}+\Delta {C}_{{\rm{s,\tau }}}{\rm{(t)}} = {\tau }_{{\rm{s,0}}}\ \Delta {R}_{{\rm{h}}}{\rm{(t)}}+{R}_{{\rm{h,0}}}\ \Delta {\tau }_{{\rm{s}}}{\rm{(t)}}$$

Hence, the equation for the component of soil carbon change due to the change in *τ*_s_ is:

4 $$\Delta {C}_{{\rm{s,\tau }}}={R}_{{\rm{h,0}}}\ \Delta {\tau }_{{\rm{s}}}$$

In this study we use *R*_h_ from the reference period (‘present day’), which we call *R*_h,0_, to allow us to investigate the response of Δ*C*_s,τ_ as a result of the response of *τ*_s_ to climate change.

### Modelled future temperature

The proof of principle figure (Fig. 3b) considers ΔC_s,τ_ between the end of the 21st century (2090–2100), for each future SSP scenario (SSP126, SSP245, SSP585)^22^ or equivalent future RCP scenario (RCP2.6, RCP4.5 and RCP8.5)^23^, and our reference period from the historical simulation (1995–2005), for each CMIP6 ESM and CMIP5 ESM, respectively.

To consider specific °C of global warming (Fig. 4), the future spatial temperature profiles at these specific global mean warming levels, for example: 1 °C, 2 °C and 3 °C global mean warming, were calculated as follows. The temperature change is calculated from our reference period (1995–2005), and then a 5-year rolling mean of global mean temperature is taken to remove some of the interannual variability. Once the year that the given temperature increase has been reached is obtained, a time average including −5 and +5 years is taken, and the spatial temperature distribution of that model averaged over the deduced time period is used for the calculations of future *τ*_s_.

### Anomaly correction for future temperature projections

To remove uncertainty due to errors in the models’ historical simulation, a spatial future temperature anomaly was projected using each model and each respective future SSP/RCP scenario separately. To calculate this, the temperature at the reference time frame (1995–2005), which overlaps the WFDEI observational temperature data time frame (2001–2010), is subtracted from the future temperature profile for each model (as calculated above), to calculate the temperature change. Then, this temperature anomaly is added onto the observational temperature dataset to give a model-derived future ‘observational’ temperature for each model.

### Proof of concept for our method

Our method relies on the idea that the spatial temperature sensitivity can be used to project and constrain the temporal sensitivity of *τ*_s_ to temperature, and subsequently global warming. To test the robustness of this method, Δ*C*_s,τ_ calculated using model Δ*τ*_s_, and temperature sensitivity relationship-derived Δ*τ*_s_, are compared.

The change in soil carbon turnover time (Δ*τ*_s_) was either calculated using model output data to obtain model-derived Δ*τ*_s_ as follows:

5 $$\Delta {\tau }_{{\rm{s}}}={\tau }_{{\rm{s}}}^{{\rm{f}}}-{\tau }_{{\rm{s}}}^{{\rm{h}}}$$

where,

6 $${\tau }_{{\rm{s}}}={C}_{{\rm{s}}}/{R}_{{\rm{h}}}$$

Or calculated using the derived quadratic $\mathrm{log}\,{\tau }_{{\rm{s}}}$-temperature relationships to obtain relationship-derived Δ*τ*_s_, which is based on the following equation:

7 $$\Delta {\tau }_{{\rm{s}}}=\exp ({\rm{p}}({T}^{{\rm{f}}}))-\exp ({\rm{p}}({T}^{{\rm{h}}}))$$

where, *T* is near surface air temperature, and *T*^f^ represents a future temperature, and *T*^h^ represents historical (present day) temperature from our reference period (1995–2005). The exponentials ($\exp$) are taken to turn $\mathrm{log}\,{\tau }_{{\rm{s}}}$ values to *τ*_s_ values. p(*T*) represents the quadratic $\mathrm{log}\,{\tau }_{{\rm{s}}}$-temperature relationship as a function of temperature to obtain our estimated $\mathrm{log}\,{\tau }_{{\rm{s}}}$.

These Δ*τ*_s_ values are then put back into the Eq. (4) (with model-specific *R*_h,0_) to obtain the corresponding Δ*C*_s,τ_ values. The proof of principle figure (Fig. 3b) investigates the robustness of our method, where projections of model and relationship-derived values of Δ*C*_s,τ_ are compared, and an *r*^2^ value of 0.90 is obtained. The correlation of the data was also tested when investigating different levels of global mean warming to obtain the constrained values (Fig. 4). The *r*^2^ values for were as follows: 1 °C is 0.84, 2 °C is 0.87 and 3 °C is 0.87.

### Calculating constrained values

To obtain the constrained values of Δ*C*_s,τ_, the model-derived future ‘observational’ temperature for each model is used together with the observational derived $\mathrm{log}\,{\tau }_{{\rm{s}}}$-temperature relationship, to project values for future *τ*_s_. Then this together with relationship-derived historical *τ*_s_ deduced using the observational temperature dataset, can be used to calculate Δ*τ*_s_. Finally global Δ*C*_s,τ_ can be obtained by multiplying Δ*τ*_s_ by the observational dataset for *R*_h,0_ (using Eq. (4)), and then calculating a weighted-global total. As each model-derived future ‘observational’ temperature is considered separately, we obtain a range of projected observational-constrained Δ*C*_s,τ_ values.

We have now obtained a set of *x* and *y* values, corresponding to the relationship-derived and modelled values of Δ*C*_s,τ_, respectively, for each ESM. Where we have an *x* and *y* value for each model, representing the modelled Δ*C*_s,τ_ (*y* values), and the model specific relationship-derived Δ*C*_s,τ_ (*x* values). We also have an *x*_obs_ value representing the mean observational-constrained Δ*C*_s,τ_ value, and a corresponding standard deviation due to the uncertainty in the modelled spatial profiles of future temperatures. We follow the method used in Cox et al. 2018, which can be seen in the ‘Least-squares linear regression’ section and the ‘Calculation of the PDF for ECS’ section of the methods from this study^32^. Using this method, we obtain an emergent relationship between our *x* and *y* data points, which we can use together with our x_obs_ and corresponding standard deviation to produce a constraint on our *y*-axis. This is shown in Fig. 4a. From this we obtain a constrained probability density function on Δ*C*_s,τ_, with a corresponding uncertainty bounds which we consider at the 68% confidence limits (±1 standard deviation). Figure 4b show the probability density functions representing the distribution of the range of projections, before and after the constraint.

This method allows us to calculate a constrained probability density function on Δ*C*_s,τ_ at each °C of global mean warming, using the data seen in Fig. 4a for 2 °C warming, and our corresponding constrained values for 1 °C and 3 °C warming. Figure 4c shows the resultant constrained mean value of Δ*C*_s,τ_ obtained for each °C of global mean warming, and the corresponding uncertainty bounds at the 68% confidence limits (±1 standard deviation).

### Calculating effective q_10_ for change in soil carbon

Simple models of soil carbon turnover are often based on just a q_10_ function, which means that *τ*_s_ depends on temperature as follows:

8 $${\tau }_{{\rm{s}}}={\tau }_{{\rm{s,0}}}\exp {\rm{((-0.1}}\,\mathrm{log}\,{{\rm{q}}}_{{\rm{10}}}{\rm{)}}\Delta T{\rm{)}}$$

We compared the results for Δ*C*_s,τ_ that would be derived from a simple q_10_ function with our emergent constraint results for Δ*C*_s,τ_, to estimate an effective q_10_ sensitivity of heterotrophic respiration.

To do this, we can obtain an equation for Δ*τ*_s_ derived from Eq. (8). This is done by considering the following, where *τ*_s,0_ is an initial *τ*_s_, we can substitute in *τ*_s_ in temperature sensitivity form to obtain an equation for Δ*τ*_s_ in temperature sensitivity form:

9 $$\Delta {\tau }_{{\rm{s}}}={\tau }_{{\rm{s}}}-{\tau }_{{\rm{s,0}}}$$

10 $$\Delta {\tau }_{{\rm{s}}}={\tau }_{{\rm{s,0}}}\exp {\rm{((-0.1}}\,\mathrm{log}\,{{\rm{q}}}_{{\rm{10}}}{\rm{)}}\Delta T{\rm{)}}-{\tau }_{{\rm{s,0}}}$$

Then, we can substitute this Δ*τ*_s_ into Eq. (4) and simplify to obtain an equation relating Δ*C*_s,τ_ and Δ*T*:

11 $$\Delta {C}_{{\rm{s,\tau }}}={R}_{{\rm{h,0}}}{\tau }_{{\rm{s,0}}}[\exp ((-0.1\,\mathrm{log}\,{{\rm{q}}}_{10})\Delta T)-1]$$

12 $$\Delta {C}_{{\rm{s,\tau }}}={C}_{{\rm{s,0}}}[\exp ((-0.1\,\mathrm{log}\,{{\rm{q}}}_{10})\Delta T)-1]$$

This equation was used to calculate different Δ*C*_s,τ_–Δ*T* sensitivity curves based on different values on q_10_, for example q_10_ = 2, with different amounts of global mean warming to represent Δ*T*, and initial observational soil carbon stocks *C*_s,0_. These curves can be seen on Figs. 1b and 4c. Note that there is no direct relationship between the effective q_10_ for soil carbon change shown in Figs. 1b and 4c, and the spatial *τ*_s_–T relationships in Fig. 3a. Our q_10_ value is an effective q_10_ value that indicates the sensitivity of global soil carbon (in the top 1 metre) to global mean temperature.

## Supplementary information

Supplementary Information

## Data Availability

The datasets analysed during this study are available online: CMIP5 model output [https://esgf-node.llnl.gov/search/CMIP5/], CMIP6 model output [https://esgf-node.llnl.gov/search/cmip6/], The WFDEI Meteorological Forcing Data [https://rda.ucar.edu/datasets/ds314.2/], CARDAMOM Heterotrophic Respiration [https://datashare.is.ed.ac.uk/handle/10283/875], MODIS Net Primary Production [https://lpdaac.usgs.gov/products/mod17a3v055/], Raich et al. 2002 Soil Respiration [https://cdiac.ess-dive.lbl.gov/epubs/ndp/ndp081/ndp081.html], Hashimoto et al. 2015 Heterotrophic Respiration [http://cse.ffpri.affrc.go.jp/shojih/data/index.html], and the datasets for observational Soil Carbon [https://github.com/rebeccamayvarney/soiltau_ec].

## Code availability

The Python code used to complete the analysis and produce the figures in this study is available in the following online repository [https://github.com/rebeccamayvarney/soiltau_ec].
