# Supplementary material for: A spatial emergent constraint on the sensitivity of soil carbon turnover to global warming
Source: Nat Commun. 2020 Nov 2;11:5544. doi: 10.1038/s41467-020-19208-8 (PMC7608627; doi:10.1038/s41467-020-19208-8)
Supplement: Supplementary file 1 — Supplementary Information [file 41467_2020_19208_MOESM1_ESM.pdf]

Supplementary Information for 'A spatial emergent constraint on the sensitivity of soil carbon turnover to global warming' by Varney et al.

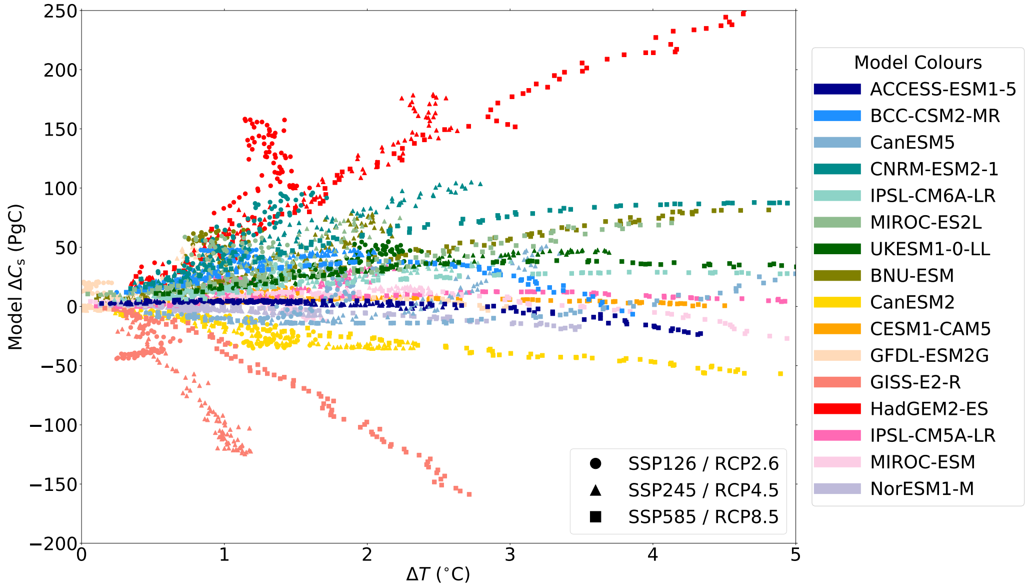

Supplementary Figure 1: **Uncertainties in future total soil carbon change.** The change in soil carbon, ΔC_{s}, against change in global mean temperatures, ΔT, diagnosed from sixteen Earth System Models (seven CMIP6 ESMs and nine CMIP5 ESMs), for three different future scenarios: SSP126, SSP245, SSP585, or RCP2.6, RCP4.5, RCP8.5 respectively.

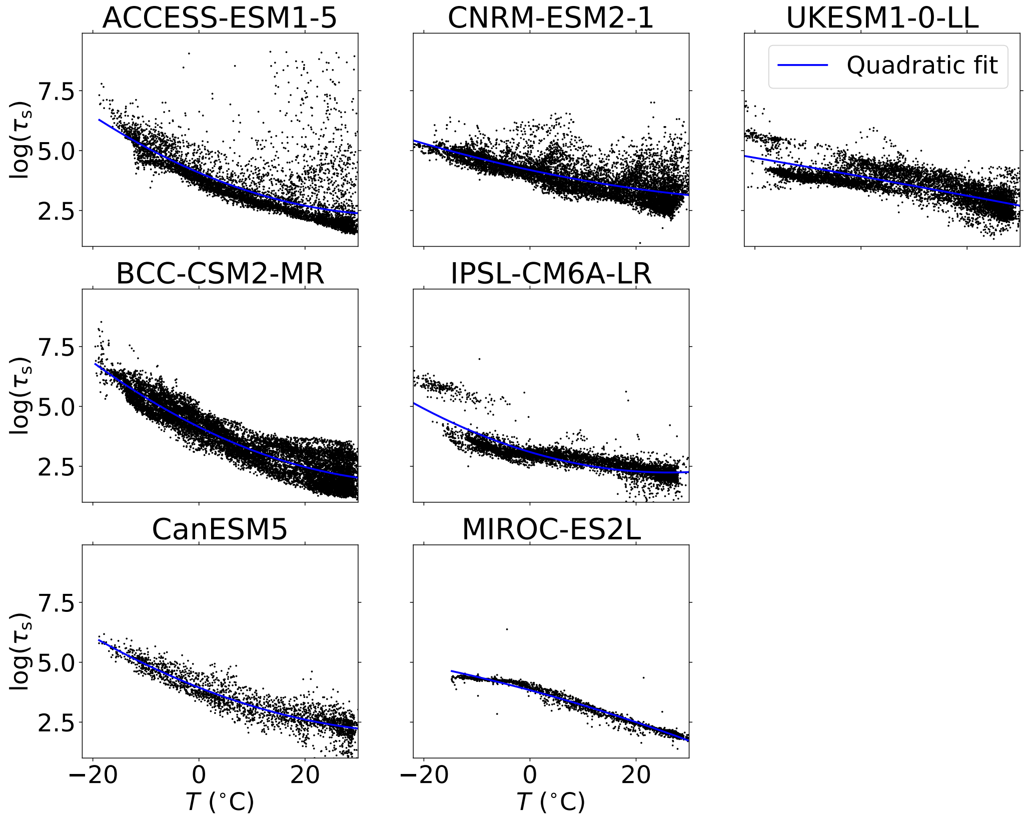

Supplementary Figure 2: **Deriving the CMIP6 model-specific** log τ_s**-temperature relationships.** Scatter plots of the relationship between soil carbon turnover, log τ_s, and near surface air temperature, T, for each CMIP6 ESM considered in our study. The black points represent the individual grid points of data, and the blue lines show the spatial log τ_s-temperature quadratic fits.

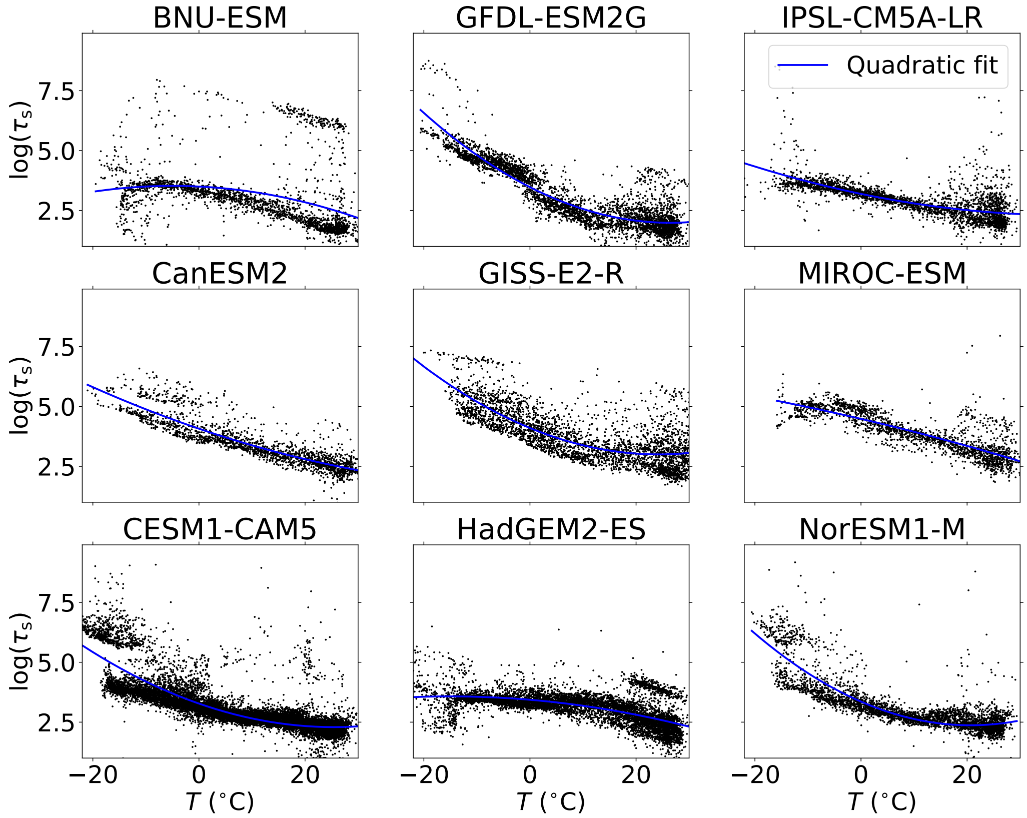

Supplementary Figure 3: **Deriving the CMIP5 model-specific** $\log \tau_\mathrm{s}$**-temperature relationships.** Scatter plots of the relationship between soil carbon turnover, $\log \tau_\mathrm{s}$, and near surface air temperature, T, for each CMIP5 ESM considered in our study. The black points represent the individual grid points of data, and the blue lines show the spatial $\log \tau_\mathrm{s}$-temperature quadratic fits.

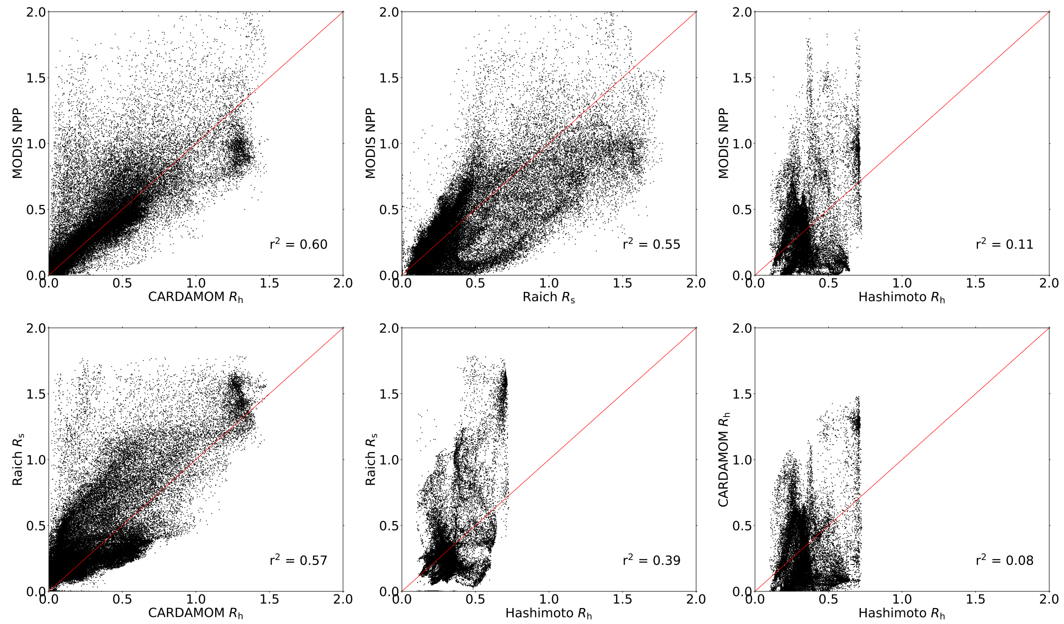

Supplementary Figure 4: **One to one comparisons of observational datasets.** Scatter plots showing one-to-one comparisons of all the observational datasets considered in this study against one another, including: CARDAMOM heterotrophic respiration (R_h) [1], MODIS net primary production (NPP) [2], Raich 2002 soil respiration (R_s) [3], and Hashimoto 2015 heterotrophic respiration (R_h) [4]. The black data points represent the spatial data for each grid point, and the red lines show a one-to-one comparison. The respective r^2 correlation coefficients are stated on each figure panel.

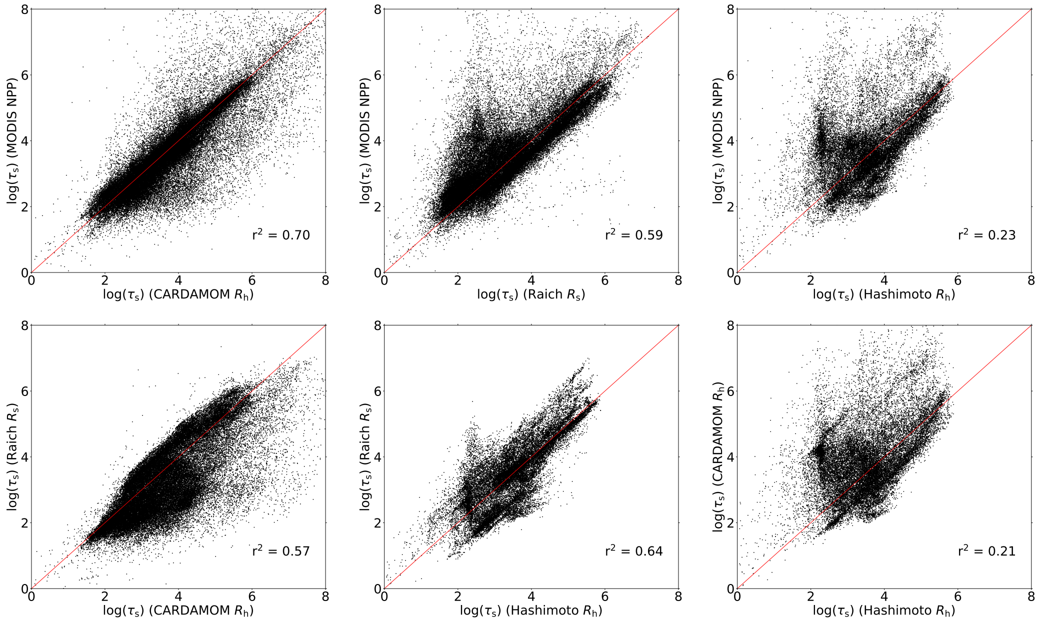

Supplementary Figure 5: **One to one comparisons of $\log \tau_s$ for each observational dataset.** Scatter plots showing one-to-one comparisons of $\log \tau_s$ calculated using each of the observational datasets considered in this study: CARDAMOM heterotrophic respiration (R_h) [1], MODIS net primary production (NPP) [2], Raich 2002 soil respiration (R_s) [3], and Hashimoto 2015 heterotrophic respiration (R_h) [4]. The black data points represent the spatial data for each grid point, and the red lines show a one-to-one comparison. The respective r^2 correlation coefficients are stated on each figure panel.

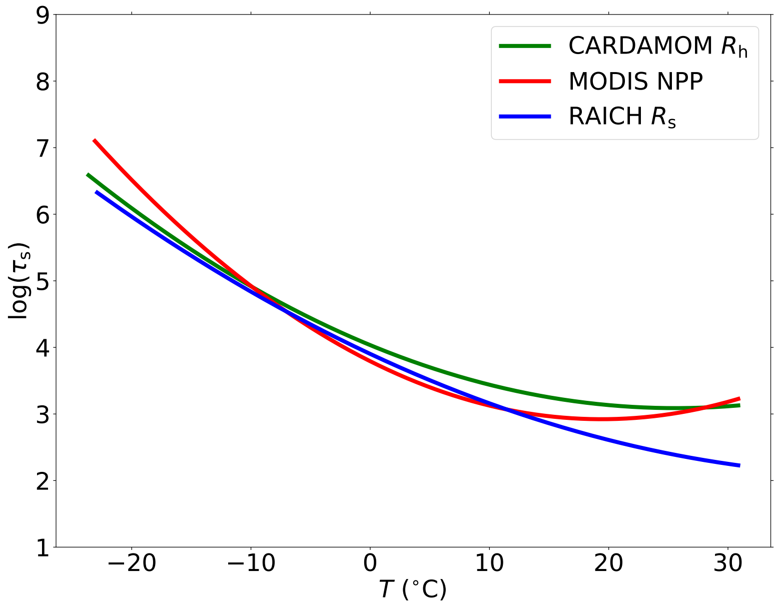

Supplementary Figure 6: **Comparison of the quadratic fits for different observational datasets.** The spatial $\log \tau_s$-temperature derived quadratic fits using our observational datasets: CARDAMOM heterotrophic respiration (R_h) [1], MODIS NPP [2], and Raich 2002 soil respiration (R_s) [3].

Supplementary References

1. Bloom, A., Williams, M. *et al.* Cardamom 2001-2010 global carbon model-data fusion (mdf) analysis (2015).

2. Zhao, M., Heinsch, F. A., Nemani, R. R. & Running, S. W. Improvements of the modis terrestrial gross and net primary production global data set. *Remote sensing of Environment* **95**, 164–176 (2005).

3. Raich, J. W., Potter, C. S. & Bhagawati, D. Interannual variability in global soil respiration, 1980–94. *Global Change Biology* **8**, 800–812 (2002).

4. Hashimoto, S. *et al.* Global spatiotemporal distribution of soil respiration modeled using a global database. *Biogeosciences* **12**, 4121–4132 (2015).
